# Report on the Medical Uses of Veratrum Viride

**Published:** 1860-11

**Authors:** A. Hard

**Affiliations:** Aurora


THE CHICAGO
MEDICAL EXAMINER

VOL. I. NOVEMBER, 1860. No. 11

### ORIGINAL COMMUNICATIONS.

### REPORT ON THE MEDICAL USES OF VERATRUM VIRIDE.

By A. HARD, M. D., of Aurora.

The medical profession have long needed some agent which could be relied upon to control the action of the heart, which should be free from the objections that apply to the use of the Lancet, and the arterial sedatives formerly employed. Feeling this want keenly, in the spring of 1852, and having read an article on the use of veratrum viride, from the pen of Dr. Norwood, published in the *North-western Medical and Surgical Journal*, I commenced its use (rather experimentally) in the treatment of Acute Pneumonia, and so completely did it fulfil the indications for which it was administered, and fill the *vacuum* among our ordinary remedies, that I have come to the conclusion to class it with our most reliable remedial agents. It always finds a place in my pocket case. In order to obtain as much practical information as possible, (and in making a report, I wish to have facts gathered at the bedside of the sick upon which to base conclusions rather than any, however, finely wrought theories), I issued two hundred circulars, and addressed one to each member of this Society, and the remainder to other physicians in this and adjoining states, asking for information upon the subject of this report. The answers to the questions proposed in the circular, with but one exception, so nearly corresponded with my own observation, that I have been confirmed in the opinion I had previously formed.

I propose to notice the questions proposed in the circular, and briefly give the conclusions to which I arrived, both from my own experience and professional correspondence.

*Question.* 1st. Have you made use of Veratrum Viride in your practice?

This was proposed for the reason, that I thought it desirable to record facts and experience rather than theories, as being more in accordance with the objects of this society, and of more practical importance to the profession at large.

*Question.* 2. In what form do you use it; (if the tincture; whose preparation?) and in what dose?

In my own experience I have been much perplexed by obtaining preparations of the veratrum viride of such variable strength, that it required considerable experimenting upon the receipt of each prescription to ascertain what was a proper dose. I have always used the tincture. Some times of my own preparation, made according to the U. S. Pharmacopœia, at other times made by druggists to order, and also, the tinct. as prepared by Tilden & Co., Keith, Norwood, Merrill, &c., &c. It is of the greatest importance that the physician be well apprised of the strength or virtue of the preparation used, as a mistake, made with so potent an agent as Veratrum, might prove fatal. I have found that Dr. Norwood's tincture corresponded to that prepared according to the U. S. Dispensatory, and that from 8 to 10 drops was a medium dose for an adult, and that half that number 4 to 5 drops to be a dose of Keith's tincture.

*Question.* 3. What are its effects?

Here, my experience corresponds with those who have answered the circular. It is the most reliable arterial sedative, most certain in its effects, and least dangerous of any with which I am acquainted. It is also emetic and diaphoretic, but secondarily so. In all cases where I have given it in sufficiently large doses to produce emesis, it first reduced the circulation below the normal standard, and the skin became uniformly bathed with perspiration. One unacquainted with its use might easily become alarmed at the great prostration and difficulty of breathing of a patient who had taken enough to produce severe vomiting, and although I consider it unnecessary to administer it in so large doses, yet when the emetic effect is produced, I find it easily controlled by ordinary alcoholic stimulants, or some of the preparations of opium, of which I prefer the tincture. Veratrum promotes the secretions from the skin and muocus membranes, and from this cause emetic doses may be dangerous particularly to young children, the secretion from the bronchial mucous membrane being so great as to produce suffocation. Therefore, although it may be administered to adults, in diseases which produce a state of high arterial excitement, without danger, it should be given to infants with the greatest care. I am pretty well satisfied that its use promotes the secretion and discharge of bile. But have not found it to act as a cathartic in any case, farther than would be expected from a general relaxation of the physical system.

*Question.* 4. In your opinion, what is its modus operandi? A solution of this question would be most desirable, but I entertained faint hopes of a satisfactory answer when it was proposed. I hoped to be able to analyze the blood taken from persons while under its influence, but have not been able to make a satisfactory test, and am as much in the dark on that point now as when I was appointed to report on this remedy.

In all cases where I have used the veratrum, sufficient time has elapsed from the administration to the apparent effects, for it to be absorbed and enter the circulation, and it is most reasonable for me to believe that it makes its impression upon the nervous system through that source, rather than upon any one set of nerves by any special tendency, choice or selection. In Vol. 6 of the *North Western Medical and Surgical Journal*, page 463, a case of poisoning is reported by Dr. J. S. Pashley, in which a man in good health took by mistake ℥ ii. of the saturated tincture of Veratrum Viride. "He staggered forward about fifteen or twenty feet, when he fell to the floor and commenced vomiting violently and complaining of distressing dyspnea and total blindness."

This case would favor the idea that the impression was made directly upon the nerves, as the medicine would hardly have had time to enter the circulation and become generally diffused through the system.

*Question.* 5. What value do you attach to it as a remedial agent?

In view of what has already been said, I regard the Veratrum Viride as being worthy of a place in our Materia Medica along with opium, which is the highest encomium I can bestow upon it.

*Question.* 6. In what diseases have you found it most useful?

I have found it most useful in diseases of an inflammatory type, such as acute Pneumonia, Rheumatism, Dysentery and Peritonitis, particularly Puerperal Peritonitis; and in treating such cases I administer a full dose when first called, so as to bring the patient under its influence as soon as possible, and in many cases it will be found as effectual in arresting the farther progress of the disease, as water is to quench fire. After the specific effects of the remedy are obtained, it should be continued in smaller doses, sufficiently large to keep the pulse at or a little below the normal standard.

But I do not propose to give my own observations to the exclusion of my correspondents, and here I would express my thanks for the number of answers to the circular which have been received, and from among them I have the following from Prof. W. H. Byford, of Chicago:

"I have used the saturated tincture for the last four years. My plan of administering it varies with the intensity and rapidity with which the disease runs its course, the possibility of interrupting its progress and the constitution of the patient. I find nervous patients more susceptible of its influence than the strong and plethoric, but not the less certainly benefited by it, because of this increased susceptibility, and any thing that decreases plethora and gives preponderance of nervous phenomena over that of the circulatory system will enhance the readiness of its action. On the contrary, anodynes will stop or very much modify its effects. The first perceptible effects are upon the circulation, though I think really secondary, reducing the pulse from tumultuous activity to calm tranquility.

The pulse, so far as I can judge, first, also becomes fuller and softer, but if the agent is still pushed further, it secondarily is rendered feeble and very soft. Its secondary effects are upon the alimentary canal producing nausea vomiting and catharsis. These symptoms are not desirable, and may be avoided by partially or totally withdrawing it after the pulse has been sufficiently modified. The general secretions including nearly or quite all of them are increased as this stage of effects are brought about, and may be very profuse particularly the hepatic, cutaneous, and urinary. Calorification next is interfered with, and coldness succeeds; probably the prostration might be easily carried to a fatal extent by still further administration of the drug. But as yet, although to those unacquainted with its peculiar operation some what alarming, I have never seen any serious results from its use. I am unable to say from observation whether it possesses abortive qualities or not, but from a remedy of such very decided perturbating character, I should fear bad effects in pregnant women, unless very closely watched. Aside from any peculiar property in this respect, my opinion is that its first effects are upon the great sympathetic nerve, and through it all the other after effects are produced. It does not seem to me to influence the cerebro spinal system in a direct manner.

My usual plan in cases of medium severity is to administer it in quantities of about one drop every hour for an adult. It may be well to give in four drop doses every four hours. If the patient takes about twenty-four drops at intervals during twenty-four hours, it will usually be enough to produce a decided effect in thirty-six or forty eight hours. Should the disease be a rapid and destructive one, I give sometimes double that quantity, soon as the pulse is controlled as much as desired, it should be lessened one half, or even more, as the case may be, but in bad cases not withdraw entirely. By careful management in this way, its influence may be prolonged to an almost indefinite time. By giving it in large doses, say two drops every hour, we may often have its effects in from twelve to twenty-four hours.

I regard it as one of our most reliable remedies, and not any more dangerous than thousands of our Sampson remedies. Opium and alcohol seem to almost instantly arouse the nervous energies of patients unduly prostrated by Veratrum Viride, and hence its effects are certainly under our control. Another fact of importance is, that we always have warning through the pulse of the approach of the graver effects, and thus are enabled to avoid them. It is essentially accumulation, so that the pulse that has resisted it for twenty-four hours, may in the next half hour or hour be very much controlled by it. I use it in all forms of fever that is likely to persist for several days, except the eruptive. I have been wary in the use of it in Scarlatina and Measles, lest it might suppress by its powerful revulsive direction to the alimentary tubes, the rash. For the treatment of their sequela, however, I think it very reliable, but it is in inflammations I esteem it most; Pneumonia, Arachnitis, Nephritis, Laryngitis, and in fact almost all inflammations, except those of the mucous membranes of the alimentary canal, and I cannot speak against it in these, for I have not used it in them. Convulsions of infancy and child-bed, Delirium Tremens, Insanity, &c., may all be benefitted by its judicious use. Hectic fever has been very favorably modified by it in some instances, also.

I have seen the pulse reduced from 160 per. minute, to 36 in the same time, after its administration, twenty-four hours in a case of recent occurrence.

In answer to the inquiries of our circular, Dr. Hiram Nance, of La Fayette, Stark Co., writes, that he has used the Veratrum Viride in his practice during the last six years. That he has used the Norwood's tincture, and gives it in doses from 6 to 7 drops every three hours, to males, and from 4 to 6 drops to females; continuing it generally until the pulse is reduced to 75 or 80 beats per minute. In regard to its effects, he says: "It is a powerful sedative; directly depressing the vital forces, acting directly on the great centre of circulation, and through the blood upon every part of the system. It possesses its sedative properties independent of any narcotic effects; subduing the arterial action in from two to five hours from the time the first dose is administered." He thinks Morphia or any of the preparations of Opium, capable of counteracting the excessive effects of the Veratrum. In regard to the value of Veratrum as a remedial agent, Dr. Nance ranks it with Calomel, Opium, and other important articles of the Materia Medica. He has found it most useful in the treatment of active inflammations, such as Pneumonia, Pleurisy, Meningitis, Rheumatism, and the more active grades of fever.

Dr. Benj. Woodward, of Galesburg, writes, that he has used the Veratrum Viride, extensively for the last three or four years; both in the form of Norwood's Tincture, and Tilden's Fluid Extract. He thinks it produces its sedative effects on the action of the heart, diminishing the frequency and force of the pulse, and the frequency of respirations, by impressions made on the par vagum nerve, and somewhat also on the sympathetic. He thinks it exhibits no narcotic qualities, and but feeble tendency to disturb the bowels; while it pretty uniformly increases the action of the skin and kidneys. In estimating its value as a remedial agent, Dr. Woodward, places it on a level with Opium, Quinine, and Calomel. The principal diseases in the treatment of which he uses the Veratrum, are the acute Phlegmasia, Apoplexy, Scarlatina, and the more sthenic grades of fever. He sometimes uses it in conjunction with Quinine, and thinks they naturally aid the action of each other.

Dr. Thos. J. Cornell, of Waterloo, states that he regards the Veratrum, as a very valuable remedy, and that he has found it "most useful in all diseases attended with much arterial excitement."

Dr. A. G. Randall, of Mechanicsburg, writes that he has used the Veratrum for eight years, chiefly in the form of Norwood's Tincture, and prefers to give it in as large doses as the stomach will bear, and "as an arterial sedative, thinks it has no equal." He also attributes to it Expectorant, Antispasmodic, and Diaphoretic Properties. The following is Dr. Randall's method of using the Veratrum in acute inflammations:

"Place the patient in a recumbent position; feet in a warm bath; fomentations to the seat of pain; and give at once from

30 to 40 drops of Tincture of Veratrum Viride, in toddy or peppermint sling, in connection with from 4 to 10 grains of Opium, or its equivalent of Morphine."

Dr. J. B. Meigs, of Manito, in answering our inquiries, says he has used the Veratrum Viride almost daily for four years, and finds it the most reliable arterial sedative we possess, promoting expectoration and diaphoresis, and also acting as a powerful antispasmodic. He uses it chiefly in the treatment of the acute Phlegmasia, Puerpural fever, and Puerpural convulsions. He successfully treated a case of the latter disease in June, 1857, and has known several other cases treated since by others with equally satisfactory results.

Dr. P. K. Guild, of Aurora, writes that he has used the Veratrum Viride in the form of Norwood's Tincture, and in doses of from two to ten drops, repeated every 3, 4 or 6 hours, Thinks it a reliable sedative, and when continued long, usually producing either emetic or cathartic action. He says, " the value I attach to it as a remedy, is what I would call second rate."

Dr. S. York, of Paris, Ill., writes that he has used the Norwood's Tincture of Veratrum Viride, in a great variety of inflammatory affections, since 1853. He regards it as a very reliable and valuable arterial sedative, rendering the use of the Lancet very rarely necessary.

Dr. T. D. Fitch, of Kewanee, states that he has used the Veratrum Viride since 1854. He prefers Norwood's Tincture to any other preparation in use, and gives it in doses of from 4 to 6 drops, repeated every three or four hours, until the desired effect is produced. He regards it as a very efficient sedative, and in large doses an emetic. In value, he regards it as worthy to be ranked with the most important articles of the Materia Medica. In his practice, especially in the treatment of the more acute Phlegmasia, he has made it supercede the use of both, Tartrate of Antimony and Potassa, and the Lancet, except in a few rare instances.

